# Optimizing CT pulmonary angiography with patient-adaptive triggering—a novel approach for a “one-stop-shop” evaluation of pulmonary and aortic vasculature

**DOI:** 10.1007/s00330-025-12148-1

**Published:** 2025-11-19

**Authors:** Gonçalo G. Almeida, Ismaiel Chikh Bakri, Natalia Leopold, Jakob Heimer, Ralf Gutjahr, Oezlem Krzystek, Maria Paslak, Tilo Niemann, André Euler

**Affiliations:** 1 Department of Radiology, Kantonsspital Baden, Affiliated Hospital for Research and Teaching of the Faculty of Medicine of the University of Zurich, Baden, Switzerland; 2 Computed Tomography, Siemens Healthineers AG, Forchheim, Germany

**Keywords:** Pulmonary artery, Aorta, Computed tomography angiography, Contrast media

## Abstract

**Objectives:**

Achieving optimal contrast opacification in CT pulmonary angiography (CTPA) is critical for diagnosing pulmonary embolism but remains challenging due to patient-specific hemodynamics. This study evaluated whether a patient-adaptive trigger delay protocol could improve vascular enhancement compared to traditional fixed-delay methods.

**Materials and methods:**

This retrospective study included 270 patients divided into three groups (*n* = 90 each): Group A (fixed delay, pulmonary trunk trigger), Group B (fixed delay, aorta trigger), and Group C (patient-adaptive delay, FAST Bolus, aorta trigger). Objective image quality was assessed using contrast-to-noise ratio (CNR). Subjective image quality, including diagnostic quality and artifact severity, was independently evaluated by two blinded radiologists.

**Results:**

The patient-adaptive protocol (Group C) yielded significantly higher CNR in the main (*p* = 0.03) and segmental lower lobe pulmonary arteries (*p* = 0.002) compared to the conventional method (Group A). Aortic CNR was significantly improved in both aorta-triggered groups (B and C) compared to Group A (*p* < 0.05). While overall subjective diagnostic quality ratings showed no statistically significant difference between groups, one of two readers rated the adaptive protocol as significantly superior to both fixed-delay methods (*p* < 0.0001).

**Conclusion:**

A patient-adaptive trigger delay with aortic monitoring significantly improves contrast opacification in the peripheral pulmonary arteries and the thoracic aorta. This approach facilitates a comprehensive “one-stop-shop” assessment, potentially enhancing diagnostic confidence for both small peripheral emboli and concurrent aortic disease.

**Key Points:**

***Question*** *Achieving optimal pulmonary artery contrast in CT pulmonary angiography (CTPA) is challenging due to variable patient-specific hemodynamics, which may potentially compromise diagnostic accuracy for pulmonary embolism.*

***Findings*** *A patient-adaptive trigger delay significantly improves contrast-to-noise ratio in peripheral pulmonary arteries and the thoracic aorta compared to traditional fixed-delay methods.*

***Clinical relevance*** *The adaptive bolus-tracking protocol enhances diagnostic quality by optimizing vessel opacification, allowing simultaneous evaluation of pulmonary and aortic pathologies, as well as potentially increasing diagnostic confidence of more peripheric pulmonary vessels.*

**Graphical Abstract:**

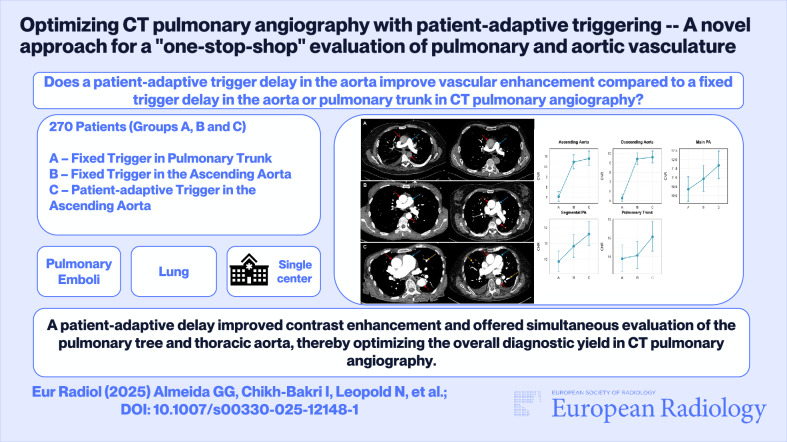

## Introduction

Pulmonary embolism (PE) is a major contributor to global cardiovascular morbidity and mortality, ranking as the third most frequent cardiovascular emergency after myocardial infarction and stroke [1, 2]. The critical importance of rapid and accurate diagnosis is underscored by an increasing prevalence of PE in the Western world and its often-non-specific clinical presentation, with symptoms including dyspnea and chest pain that may mimic other conditions such as aortic dissection [3–5]. While computed tomography pulmonary angiography (CTPA) is the established gold standard for diagnosis due to its high accuracy and availability [2], its diagnostic efficacy is fundamentally dependent on achieving optimal and homogeneous opacification of the pulmonary arteries.

Achieving this optimal enhancement is challenging, as it is influenced by patient-specific variables like cardiac output and body habitus [6, 7]. Modern CTPA protocols typically employ bolus tracking with a fixed trigger delay to standardize scan timing. This method, while simple, initiates scanning a fixed time after contrast density reaches a predefined threshold in a monitoring region (e.g., the pulmonary trunk). This “one-size-fits-all” approach, however, does not account for individual patient hemodynamics, risking suboptimal timing that can compromise diagnostic image quality [8, 9].

Recently, a patient-adaptive trigger delay algorithm (FAST Bolus, Siemens Healthineers AG) has been introduced to overcome this limitation by dynamically adjusting the scan timing based on real-time enhancement measurements. One such system has already demonstrated improved image quality and contrast homogeneity in CT angiography of the aorta [8, 10]. The feasibility and potential benefits of adaptive protocols for improving contrast enhancement timing in the diagnosis of pulmonary embolism have previously been demonstrated in preclinical animal models [11]. However, its efficacy for the specific demands of CTPA—with its unique hemodynamic considerations distinct from systemic aortic imaging—has not yet been investigated in human patients.

Therefore, the purpose of this study was to evaluate the impact of a patient-adaptive trigger delay on objective and subjective image quality in CTPA, comparing it to traditional fixed-delay protocols with trigger regions in both the pulmonary trunk and the ascending aorta.

## Materials and methods

### Patient population

This retrospective single-center study was approved by the local ethics committee. All participants signed a written informed consent. Consecutive patients ≥ 18 years of age who received a clinically indicated contrast-enhanced CTPA for suspected PE were screened for inclusion. Patients imaged before and after the clinical implementation of a patient-adaptive bolus tracking software (FAST Bolus, Siemens Healthcare AG) between March 2024 and October 2024 were included and subdivided into three groups:

1. Group A with a fixed trigger delay (6 s) and monitoring in the pulmonary trunk
2. Group B with a fixed trigger delay (6 s) and monitoring in the ascending aorta
3. Group C with a patient-adaptive delay and monitoring in the ascending aorta

Exclusion criteria included: absence of general consent (*n* = 4 in Group A and *n* = 9 in Group B), contrast injection via a central venous catheter (*n* = 10 in Group A, *n* = 3 in Group B and *n* = 1 in Group C), metallic artifacts from foreign material (*n* = 1 in Group A, *n* = 1 in Group C) and missing data on body weight/height (*n* = 1 in Group A).

The primary outcome was objective image quality (CT attenuation and contrast-to-noise ratio (CNR)) of the vessel lumen at multiple pulmonary artery locations, as well as in the ascending and descending aorta. The secondary outcome was subjective image quality.

### Scan and injection protocol

All examinations were performed on a third-generation dual-source dual-energy CT (SOMATOM Drive, Somaris 7 VB30, Siemens Healthcare AG) using our institutional scan protocol. Scan parameters were: automatic tube voltage selection (CARE kV (optimized for vascular imaging), reference kV of 120), automatic tube current modulation (CARE Dose4D, reference mAs 60), collimation of 128 × 0.6 mm, gantry rotation time of 0.28 s and pitch of 1.2. Axial images with a slice thickness of 1 mm and an increment of 1 mm were reconstructed. The contrast media protocol was identical for all groups via the antecubital vein as follows: 10 mL of 0.9%-NaCl, followed by 70 mL of non-ionic iodinated contrast media (Iopamiro 370 mgI/mL, Bracco Group) and a saline chaser of 30 mL 0.9%-NaCl. The flow rate for all injections was 4 mL/s. All scans were performed with standardized breath-hold instructions (“Inhale and hold your breath”) initiated 5 s prior to image acquisition.

Bolus tracking was initiated with a ROI placed in either the pulmonary trunk (Group A) or the ascending aorta at the level of the carina (Groups B and C), using a trigger threshold of 100 Hounsfield Units (HU). In the patient-adaptive group (Group C), the FAST Bolus software utilized a cardiovascular model to analyze real-time contrast enhancement and calculate a patient-adaptive trigger delay [12, 13]. FAST Bolus is an automated, patient-specific triggering algorithm that determines the optimal scan delay based on the upslope of the contrast bolus signal while accounting for variations in circulation, CT acquisition parameters, and injector settings, dynamically adapting the timing between bolus tracking and the start of the CTA scan [14]. Originally optimized for aortic CTA, in which time-to-peak enhancement is later than in the pulmonary bed, the software’s adaptive trigger was shifted earlier by 10 s (−10 s offset) to align with the earlier pulmonary contrast arrival.

### Objective assessment of image quality

**Fig. 1:**
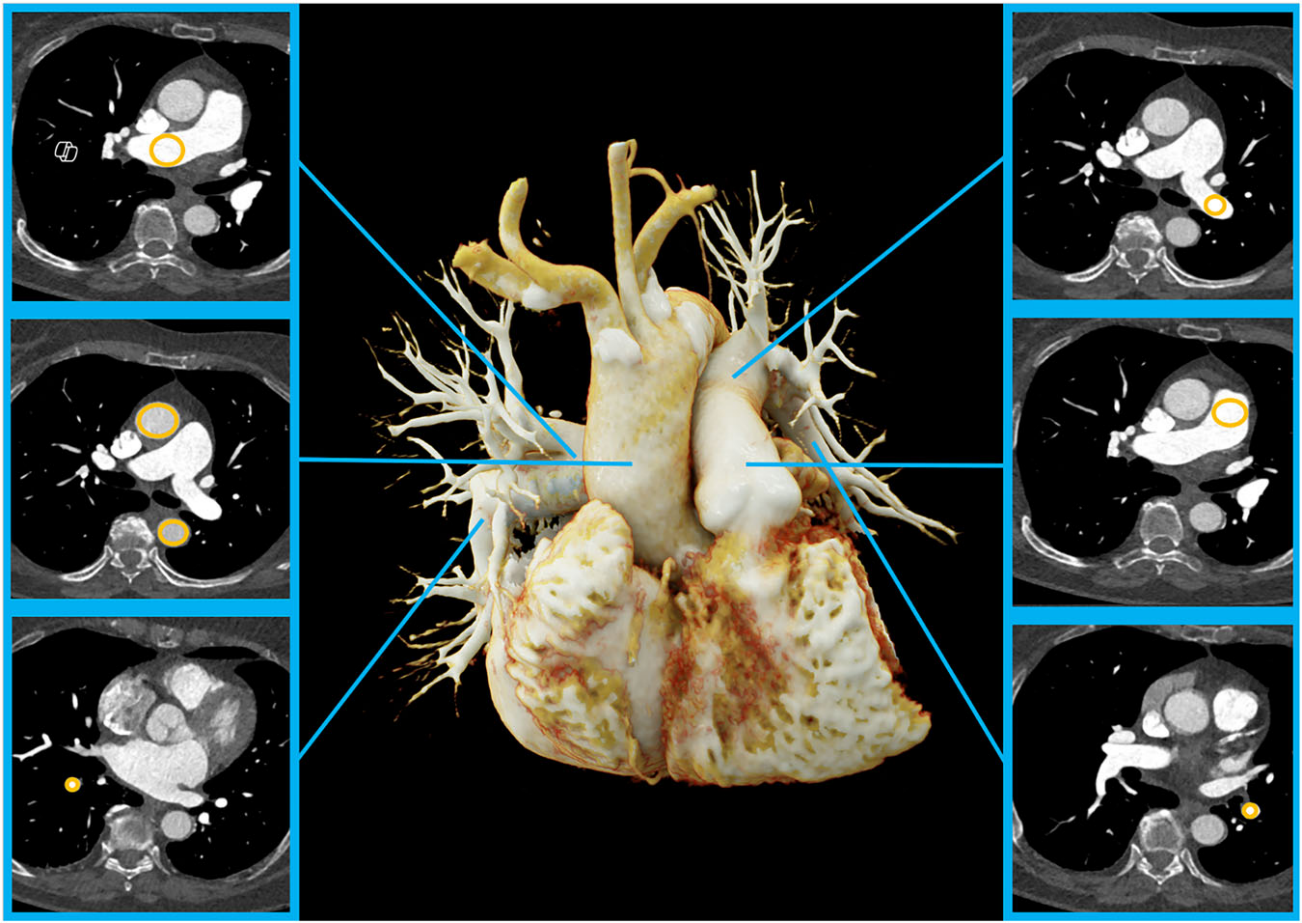
Assessment of objective image quality. Exemplary image of measurement locations in the pulmonary tree, the ascending and descending aorta. Window level: 38, Window width: 1420

CT attenuation in the vessel lumen was measured by a board-certified general radiologist with 6 years of experience by placing ROIs along the pulmonary tree, as well as in the ascending and descending aorta at the following locations: pulmonary trunk, right and left main pulmonary arteries, superior segmental pulmonary arteries of the lower lobe on each side, ascending aorta and descending aorta at the level of the origin of the right pulmonary artery (Fig. 1). ROIs were placed within the vessel lumen as large as possible while avoiding the vessel wall and calcifications. In addition, contrast-to-noise ratio (CNR) was calculated as follows:

$${CNR}=\frac{(H{U}_{{vessel}}-H{U}_{{muscle}})}{{Image\; noise}}$$

with HU_vessel_ representing the CT attenuation at the respective vessel location, HU_muscle_ the CT attenuation of the latissimus dorsi muscle and image noise the standard deviation of CT attenuation of the pulmonary trunk.

### Assessment of subjective image quality

**Fig. 2:**
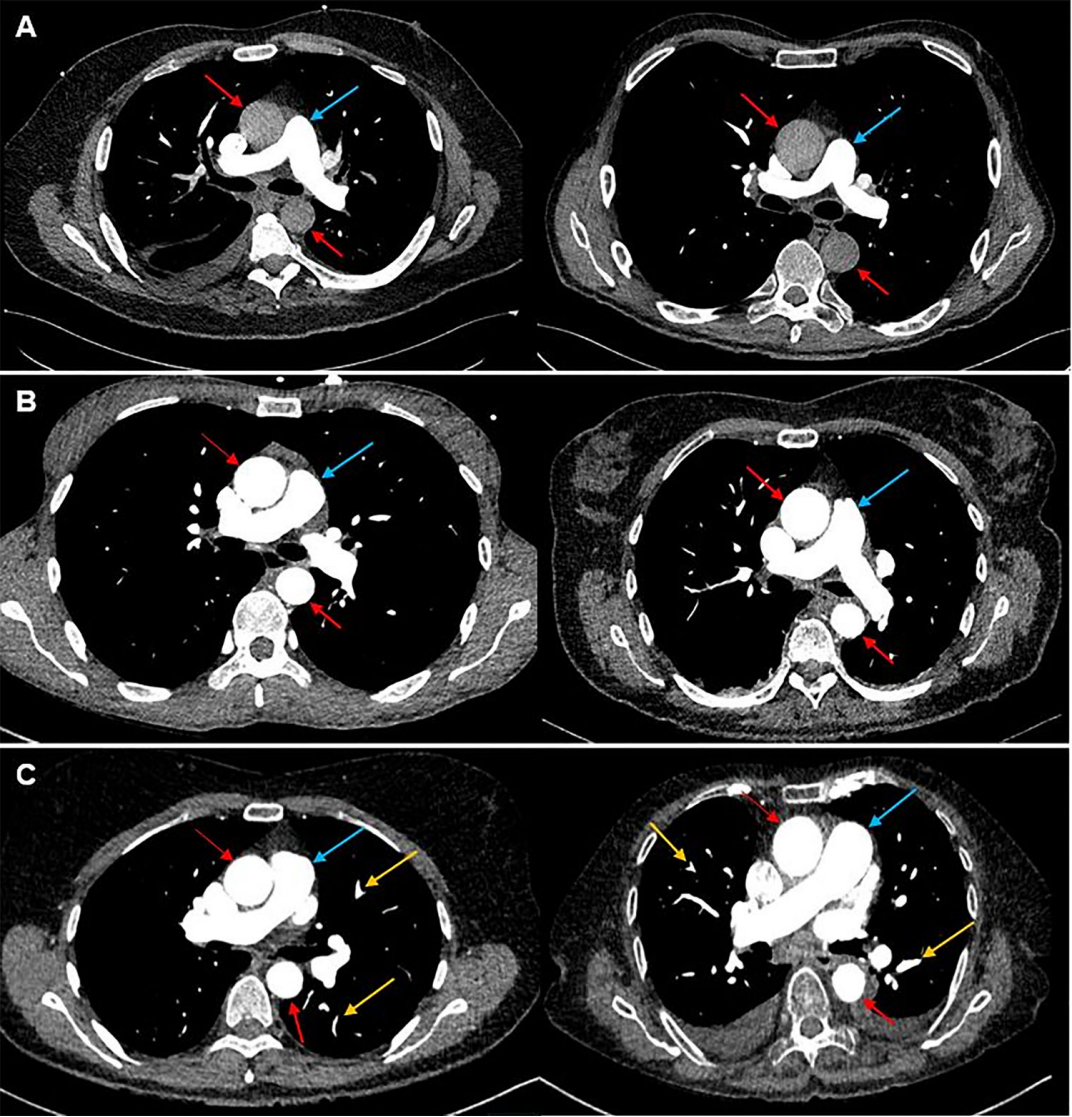
Clinical examples of CTPA. Six different patients who underwent CTPA for suspected pulmonary embolism. Triggering in the pulmonary trunk (upper row, **A**) shows only minimal enhancement in the ascending and descending aorta (red arrows). In comparison, both the aorta and the pulmonary trunk (blue arrows) show strong enhancement when triggering in the ascending aorta with either a fixed (middle row, **B**) or patient-adaptive delay (lower row, **C**). Some small peripheric segmental vessels (yellow arrows) also show strong enhancement in the patients scanned with patient-adaptive delay. Window level: 50, Window width: 350

Two board-certified radiologists with 6 and 5 years of experience, respectively, independently assessed subjective image quality for the whole pulmonary tree, from the central to the subsegmental level, with emphasis on the diagnostic confidence for detecting or excluding pulmonary embolism (Fig. 2). The readers were blinded to all patient data and triggering methods, and images were presented in a randomized order. The following 3-point Likert scale was used for diagnostic quality in accordance with international guidelines as: 1 (non-diagnostic), 2 (adequate), or 3 (excellent). Adequate image quality was defined as suboptimal depiction of the subsegmental pulmonary arteries that nevertheless allowed diagnostic assessment of pulmonary embolism (PE). In contrast, “excellent” image quality indicated optimal opacification of the subsegmental arteries with minimal artifacts. The extent of artifacts was assessed using a 5-point Likert scale (1 = none, 2 = mild, 3 = moderate, 4 = significant, 5 = severe). “Significant” artifacts were defined as those partially obscuring the evaluation of segmental pulmonary arteries, whereas “severe” artifacts were those completely preventing diagnostic assessment, including obscuration of the main pulmonary arteries. In addition, the readers had to state whether the artifact in the SVC affected the diagnostic assessment of the superior segmental pulmonary arteries of the right upper lobe.

### Statistical analysis

A prior power analysis for sample size estimation was conducted using data from 10 patients who underwent CTPA. The effect size was medium (d = 0.5) using Cohen’s (1988) criteria. With a significance criterion of α = 0.05 and power = 0.8, a minimum sample size of 83 per group was estimated. Data distribution was assessed visually, revealing a substantial right skew in the Likert items, which was analyzed using a mixed-effects model to compare Likert scores across categories and readers. Quantitative CT attenuation metrics were analyzed using mixed-effects models with CNR as the dependent variable, incorporating ‘trigger location’ and ‘measurement location’, and their interaction as fixed effects. Patient-specific random intercepts and random slopes for ‘trigger location’ were included. Age and kV served as covariates. Post hoc tests compared CNR between the aorta and pulmonary tree across measurement locations for each group. All results were considered significant at *p* < 0.05. Continuous variables are presented as mean ± standard deviation (SD) and Likert items as median and interquartile range (IQR). Patient data was compared using independent *t*-tests or ANOVA as appropriate. Analyses were conducted using the R software (v4.3.0).

## Results

### Patient characteristics

Patient characteristics are summarized in Table 1.

**Table 1:** Patient characteristics

	Group A	Group B	Group C	*p*-value
Patient characteristics				
Age (years)	65.1 ± 18.2	63.8 ± 18.8	65.8 ± 18.2	0.76
Sex				
Female, *n* (%)	42 (46.7)	46 (51.1)	52 (57.8)	
Male, *n* (d%)	48 (53.3)	44 (48.9)	38 (42.2)	
BMI (kg/m^2^)	26.8 ± 6.7	26.8 ± 6.3	26.7 ± 5.1	0.99
Scan parameters				
Delay time (s)	6	6	5 (2–6)	
CTDI_vol_ (mGy)	2.9 ± 1.6	2.8 ± 1.6	2.8 ± 1.5	0.94
DLP (mGy × cm)	101.3 ± 57.9	98.8 ± 58.5	92.3 ± 39.0	0.50
Tube voltage (kV)	90 (70–120)	90 (70–110)	90 (70–100)	0.43

Data are mean ± standard deviation or absolute values with percentages in brackets. Tube voltage is noted as median and range in brackets

A total of 270 patients (90 patients in each group) were included. There was no statistically significant difference in age (65.1 ± 18.2 years, 63.8 ± 18.8 years, 65.8 ± 18.2 years for Group A, B and C, respectively; *p* = 0.76), body mass index (mean BMI 26.8 ± 6.7 kg/m^2^, 26.8 ± 6.3 kg/m^2^, 26.7 ± 5.1 kg/m^2^ for Group A, B, and C, respectively; *p* = 0.99) or tube voltage (median tube voltage of 90 kV for each group; *p* = 0.33) among the groups. Furthermore, there was no significant difference in radiation dose among the groups (volume CT dose index (CTDI_vol_): 2.9 ± 1.6 mGy, 2.8 ± 1.6 mGy, 2.8 ± 1.5 mGy for Group A, B and C, respectively; *p* = 0.94 and dose length product (DLP): 101 ± 58 mGy × cm, 99 ± 59 mGy × cm, 92 ± 39 mGy × cm for Group A, B and C, respectively; *p* = 0.50). The patient-adaptive trigger delay chosen by the software ranged between 2 s and 6 s with a mean of 5 s ± 0.4 s.

### Objective image quality assessment

**Fig. 3:**
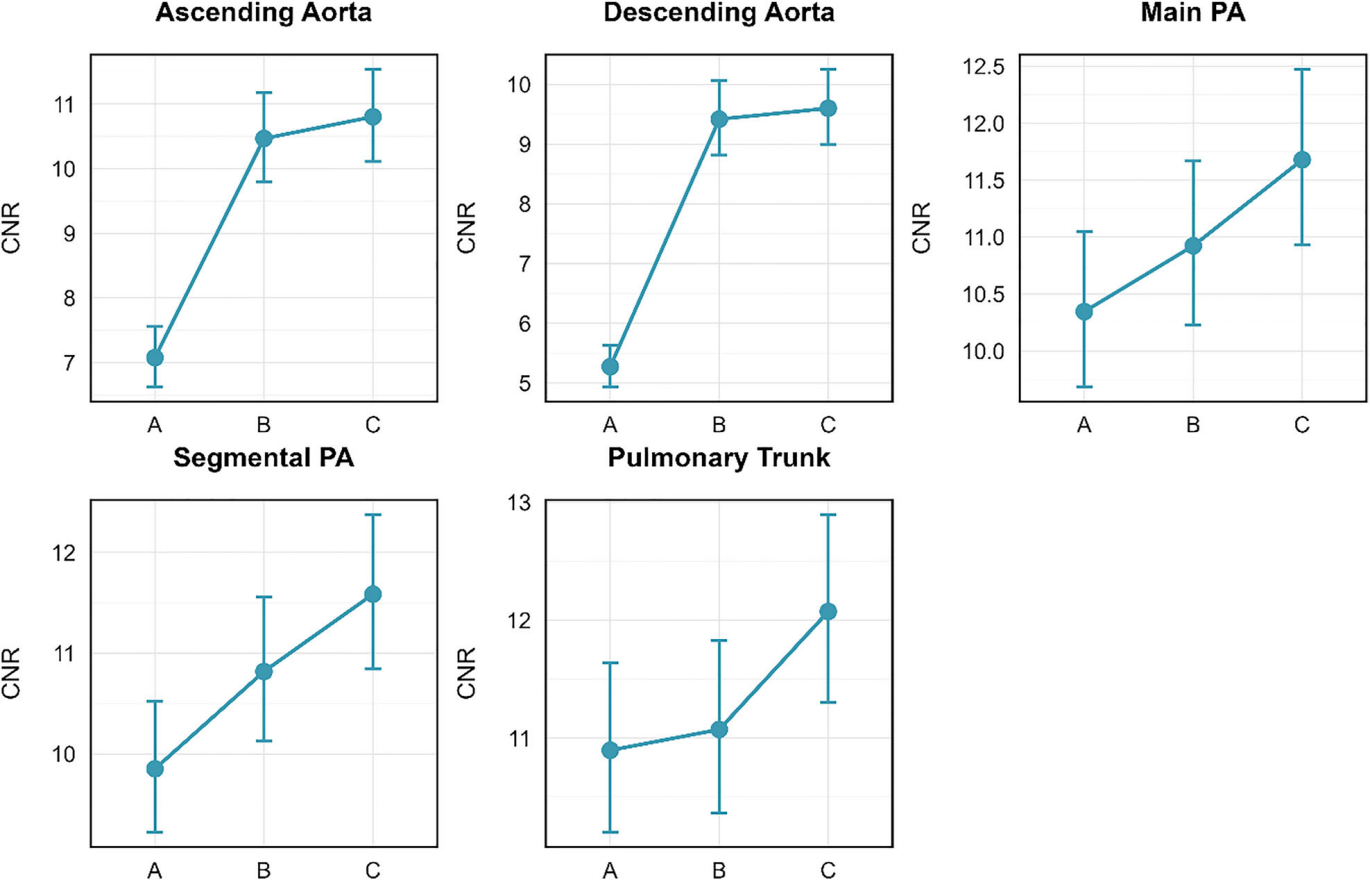
CNR as a function of scan approach and vessel location. Graphs indicate mean CNR and standard deviation among Group A, B C for different measurement locations along the pulmonary tree and thoracic aorta. The plots show mean estimates corresponding to 95% confidence intervals. Please note the highest CNR at all locations for Group C

Detailed results are summarized in Tables 2 and 3 and Fig. 3.

**Table 2:** CT attenuation at different vessel locations

Measurement location	CT attenuation (HU)	CNR
Ascending aorta		
Group A	321 ± 36	7.8 ± 3.4
Group B	447 ± 38	11.0 ± 3.4
Group C	442 ± 38	11.3 ± 3.0
Descending aorta		
Group A	264 ± 35	6.2 ± 3.3
Group B	407 ± 36	9.9 ± 3.1
Group C	396 ± 36	10.0 ± 2.8
Pulmonary trunk		
Group A	449 ± 35	11.4 ± 4.0
Group B	477 ± 36	11.8 ± 4.0
Group C	495 ± 36	12.7 ± 3.7
Right main pulmonary artery		
Group A	430 ± 39	10.8 ± 3.6
Group B	473 ± 39	11.7 ± 4.0
Group C	483 ± 40	12.3 ± 3.5
Left main pulmonary artery		
Group A	425 ± 37	10.7 ± 3.6
Group B	470 ± 38	11.6 ± 4.0
Group C	478 ± 37	12.2 ± 3.6
Right segmental lower lobe pulmonary artery		
Group A	412 ± 32	10.3 ± 3.8
Group B	468 ± 35	11.5 ± 4.1
Group C	481 ± 34	12.2 ± 3.7
Left segmental lower lobe pulmonary artery		
Group A	414 ± 33	10.4 ± 3.8
Group B	474 ± 34	11.7 ± 4.5
Group C	481 ± 35	12.2 ± 3.8

Data are mean ± standard deviation

**Table 3:** Post hoc pairwise comparisons of CNR by location and group

Measurement location	Ratio	Standard error	*p*-value
Ascending aorta*			
A/B	0.68	0.03	0.000*
A/C	0.65	0.03	0.000*
B/C	0.97	0.05	0.505
Descending aorta*			
A/B	0.56	0.03	0.000*
A/C	0.55	0.03	0.000*
B/C	0.98	0.05	0.686
Main PA*			
A/B	0.95	0.05	0.254
A/C	0.89	0.04	0.033*
B/C	0.94	0.04	0.254
Segmental PA			
A/B	0.91	0.04	0.100
A/C	0.85	0.04	0.002*
B/C	0.93	0.04	0.149
Pulmonary trunk			
A/B	0.98	0.05	0.736
A/C	0.90	0.04	0.093
B/C	0.92	0.04	0.137

Table presenting pairwise comparisons of CNR ratios (A/B, A/C, B/C) between the three groups as a function of anatomical site. Ratios below 1.0 indicate inferior CNR of the first group in the comparison. Please note that Group C showed significantly higher CNR in both the main and segmental pulmonary arteries compared to Group A, indicating improved pulmonary vascular contrast. CNR in the ascending and descending aorta was significantly lower in Group A compared to both Group B and C. Asterisks (*) and in bold indicate statistically significant differences (*p* < 0.05)

For instance, mean CT attenuation was 449 ± 35 HU, 477 ± 36 HU, 495 ± 36 HU in the pulmonary trunk and 321 ± 36 HU, 447 ± 38 HU, 442 ± 38 HU in the ascending aorta for Group A, B, and C, respectively. CNR was 11.4 ± 4 HU, 11.8 ± 4 HU, 12.7 ± 3.7 HU in the pulmonary trunk, 10.3 ± 3.8, 11.5 ± 4.1, 12.2 ± 3.7 in the right segmental lower lobe pulmonary artery, 7.8 ± 3.4 HU, 11 ± 3.4 HU, 11.3 ± 3 HU in the ascending aorta, and 6.2 ± 3.3 HU, 9.9 ± 3.1 HU, 10 ± 2.8 HU in the descending aorta for Group A, B, and C, respectively. The linear mixed-effects model evaluated the effects of trigger method and measurement location on CNR. Post hoc comparisons of estimated marginal means revealed that CNR of the pulmonary trunk was not inferior in the patient-adaptive delay group (Group C) when compared to the fixed delay group with triggering in the pulmonary trunk (Group A) (*p* = 0.093). In the main pulmonary arteries and in the segmental lower lobe pulmonary arteries, CNR was significantly higher in Group C than in Group A (*p* = 0.03 and *p* = 0.002, respectively). There was no statistically significant difference in CNR of the pulmonary arteries between Group B and Group C (*p* = 0.505 for the ascending aorta; *p* = 0.686 for the descending aorta). CNR in the ascending and descending aorta was significantly higher in Group B and C as compared to Group A (all *p* < 0.05).

### Subjective image quality assessment

**Fig. 4:**
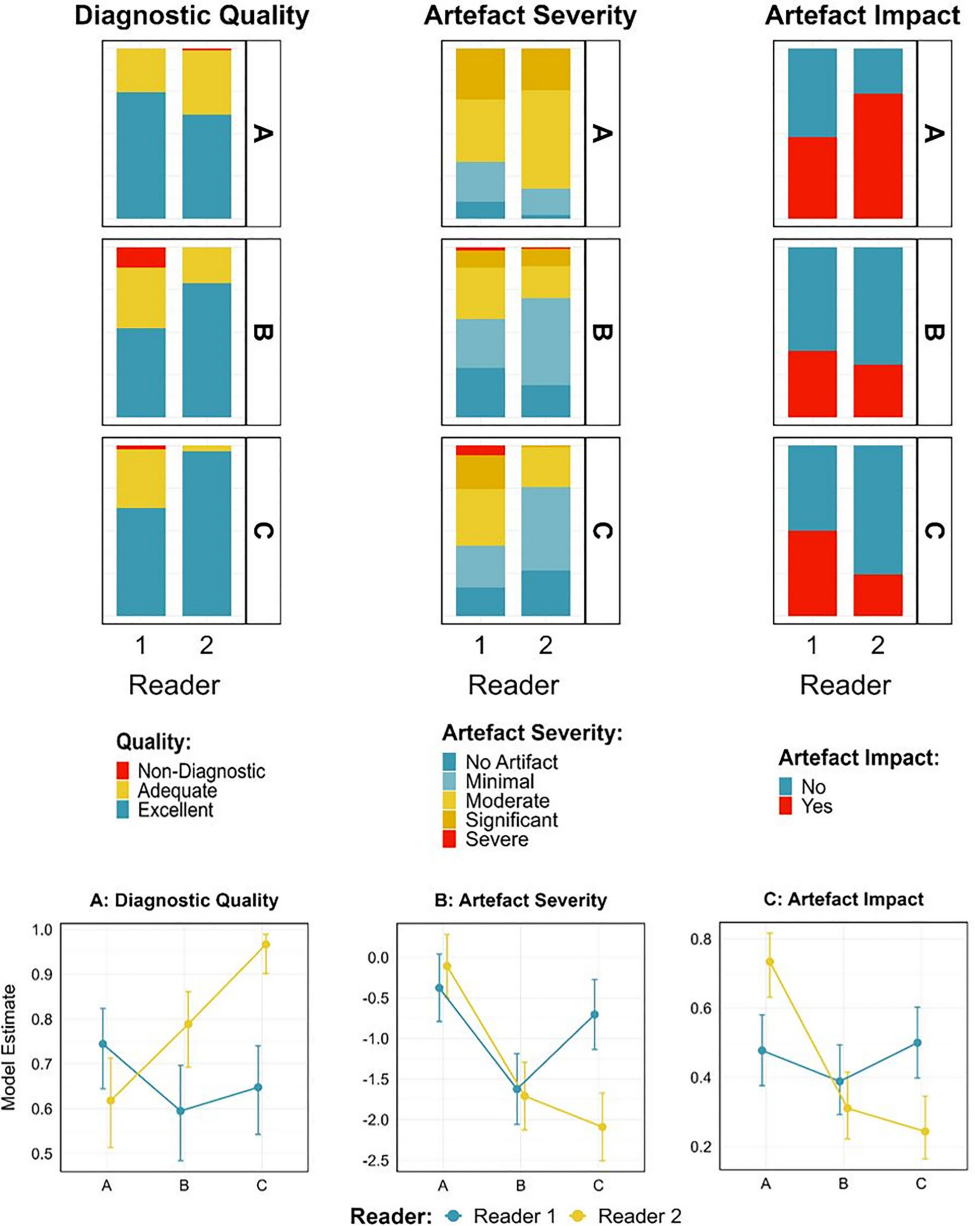
Subjective image quality. Box diagrams (above) and linear plots (below) depicting the subjective evaluation of diagnostic quality of pulmonary embolism CT (left), the amount of artifact in the SVC (middle) and whether these artifacts affect the diagnostic evaluation of the right superior segmental upper lobe arteries. (right) for Reader 1 (blue plot) and Reader 2 (yellow plot) across groups A, B and C. Please note the increase in diagnostic quality and the reduction in the effect of the SVC artifact in Group C for Reader 2

Results are summarized in Tables 4 and 5 and Fig. 4.

**Table 4:** Subjective image quality

	Reader 1			Reader 2		
	Group A	Group B	Group C	Group A	Group B	Group C
Diagnostic quality						
1 (non-diagnostic)	0 (0%)	11 (12%)	2 (2%)	1 (1%)	0 (0%)	0 (0%)
2 (adequate)	23 (26%)	32 (36%)	31 (34%)	34 (38%)	19 (21%)	3 (3%)
3 (excellent)	67 (74%)	47 (52%)	57 (63%)	55 (61%)	71 (79%)	87 (97%)
Artifact severity						
1 (no artifact)	9 (10%)	26 (29%)	15 (17%)	2 (2%)	17 (19%)	24 (27%)
2 (minimal artifact)	21 (23%)	26 (29%)	22 (24%)	14 (16%)	46 (51%)	44 (49%)
3 (moderate artifact)	33 (37%)	27 (30%)	30 (33%)	52 (58%)	17 (19%)	21 (23%)
4 (significant artifact)	27 (30%)	9 (10%)	18 (20%)	22 (24%)	9 (10%)	1 (1%)
5 (severe artifact)	0 (0%)	2 (2%)	5 (6%)	0 (0%)	1 (1%)	0 (0%)
Artifact impact						
Yes (%)	43 (48%)	35 (39%)	45 (50%)	66 (73%)	28 (31%)	22 (24%)
No (%)	47 (52%)	55 (61%)	45 (50%)	24 (27%)	62 (69%)	68 (76%)

Data are presented as absolute numbers with percentages in brackets. Artifact severity was assessed in the superior vena cava and artifact impact in the superior segmental pulmonary arteries

**Table 5:** Post hoc pairwise comparisons of subjective image assessment by reader and group

Subjective outcomes			
	Ratio	Standard error	*p*-value
**Diagnostic quality**			
Reader 1			
A/B	1.98	0.66	0.119
A/C	1.58	0.52	0.324
B/C	0.80	0.26	0.482
Reader 2			
A/B	0.43	0.15	0.013*
A/C	0.06	0.03	0.000*
B/C	0.13	0.08	0.003*
**Artifact severity**			
Reader 1			
B/A	−1.25	0.29	0.000*
C/A	−0.33	0.28	0.247
C/B	0.92	0.29	0.003*
Reader 2			
B/A	−1.60	0.27	0.000*
C/A	−1.98	0.27	0.000*
C/B	−0.38	0.26	0.150
**Artifact impact**			
Reader 1			
A/B	1.44	0.44	0.463
A/C	0.91	0.27	0.766
B/C	0.64	0.19	0.409
Reader 2			
A/B	6.14	2.10	0.000*
A/C	8.57	3.07	0.000*
B/C	1.40	0.47	0.320

Table summarizing the pairwise comparisons (A/B, A/C, B/C) between the three groups for key subjective outcomes, including overall diagnostic image quality, severity of artifacts in the superior vena cava (SVC), and artifact-related diagnostic impairment of the superior segmental pulmonary arteries. Please note that, for artifact severity, estimates were inverted so that positive values indicate more artifacts. Results are presented as ratio estimates (or differences), standard errors, and *p*-values. Please note that Group C (patient-adaptive delay) showed significantly better image quality than Groups A and B for Reader 2. Group B (fixed delay in ascending aorta) resulted in the fewest SVC artifacts for both readers. Asterisks (*) and in bold indicate statistically significant differences (*p* < 0.05)

For Reader 1, no statistically significant difference in diagnostic quality was found among the groups. For Reader 2, Group C showed significantly superior diagnostic image quality as compared to Group A and B (both *p* = 0.0001). Reader 1 rated 11 cases in Group B and 2 cases in Group C as non-diagnostic for PE, with the majority of cases being considered diagnostic (32% rated adequate and 63% rated excellent). Reader 2 rated only one case in Group A as non-diagnostic; otherwise, all cases were deemed as diagnostic for PE (21% rated adequate and 79% rated excellent).

The amount of SVC artifacts differed significantly between the groups. For Reader 1, Group A and C were associated with significantly more artifacts than Group B (both *p* < 0.004). No statistically significant difference was found between Groups A and C (*p* = 0.247). For Reader 2, Group A had significantly more artifacts than Group B and Group C (both *p* < 0.001). No significant difference was found between Group B and C (*p* = 0.150). Moderate to severe artifacts (scores 3–5) were found in 67%, 42%, 59% of cases for Reader 1 and in 82%, 30%, 24% of cases for Reader 2 in Groups A, B and C, respectively.

Regarding whether SVC artifacts affected the diagnostic assessment of the superior segmental pulmonary arteries, Reader 1 found no significant differences between the groups (*p* = 0.463, *p* = 0.766, *p* = 0.409), while for Reader 2, artifacts had a significantly higher impact in Group A as compared to Group B and C (both *p* < 0.001). No significant difference was found between Groups B and C (*p* = 0.320). Reader 1 reported artifact-related diagnostic limitations in 48%, 39%, and 50% of cases for Group A, B, C, respectively. Reader 2 reported such effects in 73%, 31%, and 24% of cases for Group A, B, and C, respectively.

## Discussion

This study aimed to compare the objective and subjective image quality of CTPA among patient-adaptive and traditional fixed trigger delay protocols with monitoring regions in both the pulmonary trunk and the ascending aorta. Our findings suggest that bolus tracking in the aorta with both a fixed and patient-adaptive trigger delay (Groups B and C) resulted in a similar CNR of the pulmonary trunk as compared to bolus tracking in the pulmonary trunk (Group A), while simultaneously improving CNR in the ascending and descending aorta. Furthermore, the patient-adaptive approach demonstrated significantly higher CNR in the main and segmental pulmonary arteries as compared to bolus tracking in the pulmonary trunk (Group A). The increased CNR observed in the more peripheral pulmonary arteries was a key advantage of the patient-adaptive approach. Optimal opacification of these smaller, segmental vessels is critical for diagnosing small or isolated PEs, and suboptimal enhancement is a known factor that can limit diagnostic confidence.

Several different injection and scan approaches have been investigated in the literature to improve contrast opacification and consistency in CTPA. For instance, the test bolus method involves injecting a small amount of contrast media prior to the diagnostic scan to precisely calculate the individual patient’s circulation time to peak enhancement. While effective, this technique increases the total iodine load and workflow complexity. Furthermore, a retrospective study in 200 patients undergoing CTPA demonstrated superior enhancement of both pulmonary arteries and the aorta using bolus tracking when compared to test bolus with the ROI set in the pulmonary trunk [15]. Other strategies focus on the contrast injection regimen itself, such as tailoring the total contrast volume and injection rate to the patient’s body weight, which has been shown to improve enhancement consistency, particularly in obese patients [6]. Furthermore, virtual monoenergetic images from dual-energy CT at low energy levels (e.g., 40 and 50 keV) have been shown to improve the contrast opacification in PE [16, 17]. Iterative and deep learning reconstruction algorithms demonstrated improved image quality, and some software packages use artificial intelligence to perform a “virtual” subtraction of a non-contrast image from the contrast-enhanced image to isolate and then boost the iodine signal, increasing the apparent opacification of the vessels [18–21].

CTPA protocols in the literature have predominantly applied bolus tracking or test bolus with a ROI set in the pulmonary trunk [16, 22, 23]. This may be the result of literature scarcity investigating bolus tracking in other anatomic sites, like the aorta. Our results suggest that bolus tracking in the aorta might be more beneficial due to simultaneous opacification of the pulmonary and aortic vessels as a “one-stop-shop” approach.

Patient-adaptive trigger delays have the potential to tailor scan timing to individual patient characteristics like cardiac output. A recent study by Rau et al with a large patient cohort demonstrated the impact of patient cardiac function on bolus arrival times and vessel enhancement when using bolus tracking to evaluate contrast dispersion in both CTA and CT-perfusion (CTP) [23]. Reduced cardiac output was shown to be directly correlated to increasing age within their patient group and to lead to slower contrast mixing within the cardiovascular system, resulting in delayed and increased bolus arrival. The authors suggest that CTA timing may not be optimal using bolus tracking followed by a fixed delay, underscoring the need for specialized software with a patient-adaptive trigger delay. Similarly, Sakai et al showed that body mass index correlated with increased cardiac output, which, in turn, influenced peak enhancement timing in the aorta [24]. Similarly, Hinzpeter et al, Sahin-Uzuner et al and Yuan et al have reported superior enhancement and diagnostic consistency in the aorta and head-and-neck arteries, respectively, using the patient-adaptive scan software used in this study [8, 10, 25]. A retrospective study by Damm et al reported significantly higher enhancement in the pulmonary trunk using bolus tracking with a fixed trigger delay and monitoring in the descending aorta; however, this study used various scanner types, variable contrast volumes, and did not apply patient-adaptive delays [26]. In our study, adapting the trigger delay to the patient’s cardiac output further optimizes the contrast within the most peripheric segments of the pulmonary tree, while allowing for simultaneous evaluation of the aorta.

Subjective assessment of image quality differed between the two readers. For Reader 1, there was no statistically significant difference in diagnostic image quality among the groups. For Reader 2, the patient-adaptive approach resulted in significantly higher image quality ratings as compared to the fixed-delay approaches, with 97% of scans rated as diagnostic for pulmonary embolism. Regarding artifacts due to contrast medium in the superior vena cava (SVC), both readers’ ratings differed significantly, but triggering in the aorta (Groups B and C) resulted in fewer artifacts than in the pulmonary trunk (Group A). However, their agreement on which aortic-triggered method was superior diverged. Regarding the impact of artifacts in the SVC on the evaluation of the superior segmental arteries, there was no significant difference among the groups for Reader 1, while diagnostic assessment was significantly affected in Group A for Reader 2. This suggests that moving the trigger location away from the pulmonary trunk to more distal locations can potentially reduce SVC artifacts by allowing more time for the dense contrast bolus to pass the venous inflow.

This study had several limitations. First, it was conducted retrospectively at a single center using a single CT platform of one vendor and a single scan protocol, potentially limiting the generalizability of results. Second, we did not have access to detailed patient hemodynamic data, such as cardiac output or ejection fraction. Correlating these physiological parameters with the performance of the adaptive algorithm would be a valuable next step to confirm its mechanism of action. Third, we did not apply weight-based contrast media application. Such an adaptation could result in variation in injection times and the timing required to achieve optimal contrast enhancement. Applying a patient-adaptive delay might be beneficial in such protocols. Fourth, although two experienced radiologists evaluated the images independently, their ratings differed significantly, underscoring the inherent variability of qualitative image assessment.

In conclusion, our results suggest that bolus tracking with a patient-adaptive trigger delay in the ascending aorta improved contrast enhancement in the peripheral pulmonary arteries and the thoracic aorta as compared to a conventional protocol with a fixed delay in the pulmonary trunk. An adaptive approach offers simultaneous evaluation of the pulmonary tree and thoracic aorta, thereby optimizing the overall diagnostic yield in CTPA. Nevertheless, these findings should be confirmed in future multicenter studies including scan protocols with reduced iodine load.

## Abbreviations

CTPA: Computed tomography pulmonary angiography
CNR: Contrast-to-noise ratio
CTP: CT-perfusion
DLP: Dose length product
PE: Pulmonary embolism
SVC: Superior vena cava
