# Correction: Optimizing CT pulmonary angiography with patient-adaptive triggering—a novel approach for a “one-stop-shop” evaluation of pulmonary and aortic vasculature

**DOI:** 10.1007/s00330-025-12304-7

**Published:** 2026-02-05

**Authors:** Gonçalo G. Almeida, Ismaiel Chikh Bakri, Natalia Leopold, Jakob Heimer, Ralf Gutjahr, Oezlem Krzystek, Maria Paslak, Tilo Niemann, André Euler

**Affiliations:** 1 Department of Radiology, Kantonsspital Baden, Affiliated Hospital for Research and Teaching of the Faculty of Medicine of the University of Zurich, Baden, Switzerland; 2 Computed Tomography, Siemens Healthineers AG, Forchheim, Germany

**Correction to: European Radiology**

 10.1007/s00330-025-12148-1;

published online 19 November 2025

In this article, the author’s name, Ismaiel Chikh Bakri, was incorrectly written as Ismaiel Chikh-Bakri. Furthermore, in the graphical abstract the CT image matching Figure 2 has been replaced.

The new graphical abstract is as follows:
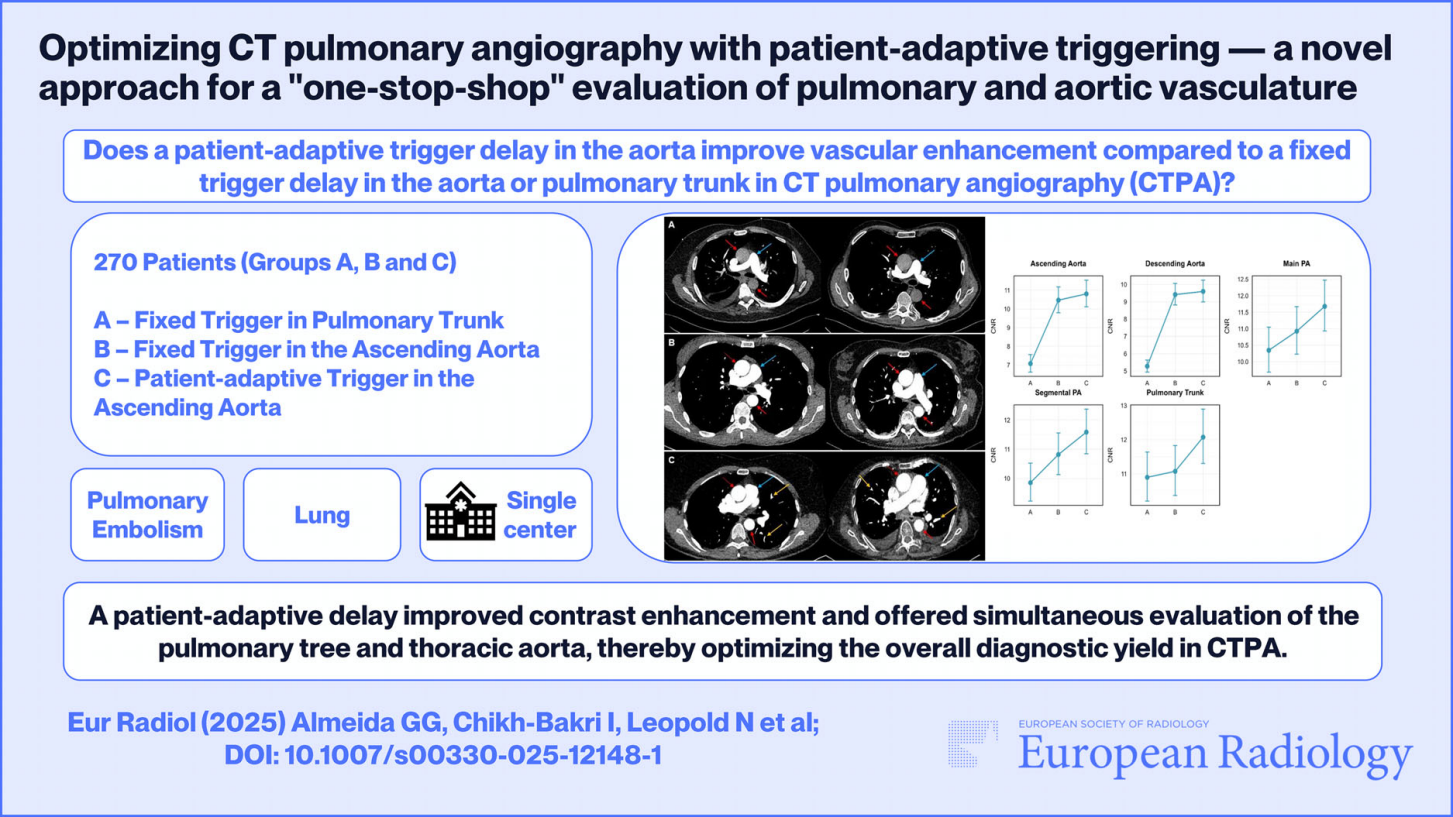


The original article has been corrected.

